# Associations Between Frailty, Sarcopenia, and Nutritional Status in Older Adults Living in Nursing Homes

**DOI:** 10.3390/nu17223574

**Published:** 2025-11-15

**Authors:** Serap İncedal Irgat, Gül Kızıltan

**Affiliations:** 1 Department of Nutrition and Dietetics, Faculty of Health Sciences, Karamanoğlu Mehmetbey University, 70100 Karaman, Türkiye; 2 Department of Nutrition and Dietetics, Faculty of Health Sciences, Başkent University, 06790 Ankara, Türkiye; gkizilta@baskent.edu.tr

**Keywords:** frailty, sarcopenia, malnutrition, nutritional status of older adults, nursing home

## Abstract

Background/Objectives: Increasing awareness of factors that put the population at high risk of frailty is essential to prevent frailty and minimize its adverse consequences. Methods: In this cross-sectional study, participants were over the age of 65 and living in nursing homes. The Edmonton Frailty Scale was used to determine frailty, the Sarcopenia Rapid Screening Test (SARC-F) was used to assess sarcopenia, and the Mini Nutritional Assessment (MNA) questionnaire and 7-day 24-h dietary recall were used to determine the nutritional status of the older adult population. Data were analyzed by SPSS 25.0 for Windows (Statistical Package for Social Sciences). Results: The frailty scale score of gender was statistically significant (*p* < 0.05). There was a statistically significant difference in sarcopenia status and malnutrition based on the distribution of the frailty status among the participants (*p* < 0.05). There was a statistically significant difference in vitamin C intake adequacy according to the distribution of frailty status among older adults (*p* < 0.05). There was a positive correlation between frailty status and sarcopenia (r = 0.773; *p* < 0.05). Frailty and nutritional status were significantly negatively correlated (r = −0.496; *p* < 0.05). There was a significant positive correlation between the sarcopenia status and malnutrition status of the participants (r = 0.489; *p* < 0.005). Conclusions: Older adults living in nursing homes are at risk for frailty syndrome, malnutrition, and sarcopenia. Evaluating older adults in terms of all these factors and implementing daily nutrition plans and support according to these results is of great importance for promoting a healthy life.

## 1. Introduction

Geriatric syndrome refers to conditions that manifest with age-related physiological and metabolic changes combined with the genetic structure of individuals, comorbidities, and typical symptoms of various etiologies (e.g., stress), occur with a combination of different clinical presentations, and do not fall under the definition of disease [1,2]. Immobility, depression, dementia, falls, sarcopenia, and frailty are examples of geriatric syndromes that reduce quality of life and increase morbidity and mortality [3].

Although there is no consensus definition of frailty, it can be defined as a clinical syndrome characterized by decreased strength, endurance, and physiological function, including dysregulation of immune, endocrine, stress, and energy regulation systems, which renders individuals vulnerable to adverse health outcomes such as physical dysfunction and increased mortality [4]. Frailty is an age-related medical syndrome of decreased functional capacity that affects approximately 10% of the population over the age of 65 [5,6]. The prevalence of frailty increases with age, female gender, low education and income, poor health, higher comorbidity, and weakness [6]. Sarcopenia has been reported in at least one in twenty individuals living in the community and in one third of the frail older adults living in nursing homes [7]. Frailty and physical decline have been found to cause emotional distress, including feelings of worthlessness and hopelessness [8]. It has been noted that frailty and depression are two distinct entities, but they show a high degree of overlap in populations categorized as both frail and depressed due to similar responses to the same stressors [9]. It is emphasized that frail elderly adults experience decreased mobility and strength and nutritional problems [3]. Half of the frail elderly are at high risk for malnutrition. Similarly, 90% of those with malnutrition are at risk for frailty or pre-frailty [10]. Reviews on frailty and nutrition have identified micro- and macronutrients, dietary patterns, diet quality, antioxidant capacity, and malnutrition as risk factors for frailty [11,12]. The InCHIANTI study of 802 individuals aged 65 years and older reported low energy intake (<21 kcal/kg) and inadequate intake of protein, vitamin E, and vitamin C as risk factors for frailty [13]. Increasing awareness of factors that put the population at high risk of frailty is essential to preventing frailty and minimizing its adverse consequences [14]. The purpose of this study was to determine the associations between frailty, sarcopenia, and nutritional status among older adults living in nursing homes.

## 2. Materials and Methods

### 2.1. Study Population

This cross-sectional study was conducted from August 2019 to March 2020, and the studied group consisted of 150 older adults from nursing homes in Karaman City Center, Turkey. The sample size of the study was calculated as 37 older adults in total with G*Power version 3.1.9.4 statistical power analysis software (90% power, effect size 0.50, and α  =  0.05). The null hypothesis assumed a zero correlation (r = 0) between the variables. Those who were under the age of sixty-five, had dementia–Alzheimer’s disease, were unable to communicate due to cognitive and mental problems, were fed by tube, or did not wish to participate were excluded from the study. We completed the study with 76 older adults (65.8% men, 34.2% women). According to the post-hoc power analysis of the 76 older adults, the power of the test was 0.99 when the correlation coefficient between the variables was 0.496 (medium level) and the error level (α) was 0.05.

### 2.2. Data Collection

#### 2.2.1. Edmonton Frailty Scale

The Edmonton Frailty Scale was developed by Rolfson et al. [15] at the University of Alberta in Canada to assess frailty in older adults. Aygör et al. [16] reported good reliability and validity for the Edmonton Frailty Scale in the Turkish population. The scale includes questions to assess the cognitive status, general health status, functional independence, social support status, medication use, nutrition, mood, and functional performance of older adults. The total score of 11 items is used in the assessment of the scale. The highest score on the scale is 17 and the lowest score is 0. An increase in the total score indicates an increase in the severity of frailty; older adults are considered “non-frail” if their score is between 0 and 4, “apparently vulnerable” if between 5 and 6, “mildly frail” if between 7 and 8, “moderately frail” if between 9 and 10, and “severely frail” if 11 or higher.

#### 2.2.2. Sarcopenia Rapid Screening Test: SARC-F

Kış [17] conducted an adaptation of the SARC-F Scale into Turkish and a validity study in individuals over 65 years of age. The scale was developed to determine the functional impact of sarcopenia on mobility and independence and consists of five sections that assess strength, walking with assistance, rising from a chair, climbing stairs, and falling. The SARC-F scores range from 0 to 10, with 0–3 representing “healthy” status and 4 and above representing “symptomatic” status. If the total score is ≥4, it is likely to indicate the presence of sarcopenia [18].

#### 2.2.3. Mini Nutritional Assessment (MNA)

The Mini Nutritional Assessment (MNA) was used to assess the nutritional status of older adults. The MNA is a simple, reliable 18-item questionnaire developed by geriatricians to assess the nutritional status of individuals [19]. Development and validation studies were conducted in France and the USA on 32 frail and healthy older adults. The validity study of the MNA in Turkey was conducted by Sarıkaya, and it can be recommended as a valid screening tool in assessing the nutritional state of Turkish geriatric subjects [20]. Each response has a numerical value and contributes to a final score of up to 30: a score of ≥24 indicates normal nutritional status; a score between 17 and 23.5, risk of malnutrition; and a score <17, malnutrition [21].

#### 2.2.4. Nutritional Status

A 24-h dietary recall form was used for 7 consecutive days to determine the nutritional status of the older adults. Standard household terms (water glass, tea glass, coffee cup, mug; tablespoon, ladle, dessert spoon; small, medium, large, etc.) and the “Food and Nutrition Photo Catalog” were used to determine amounts of food and beverages [22]. At the same time, caregivers helped by keeping records of the older adults’ daily food intake. The Nutrition Information System (BEBIS) program was used to assess the daily energy and nutrient intakes of the participants. The daily energy and nutrient intakes of the older adults were also evaluated with reference to the “Dietary Guidelines for Türkiye (TÜBER)-2015” [23].

### 2.3. Statistical Analysis of Data

The data obtained from the study were analyzed using the Statistical Package for the Social Sciences (SPSS) version 25.0 for Windows. Descriptive statistics were presented as frequency (*n*) and percentage (%) for categorical variables, and as mean and standard deviation ($\bar{X}$ ± SD) for continuous variables. The normality of the age and frailty scale scores was assessed using the Shapiro–Wilk test. For data that did not meet the assumptions of parametric tests, the Mann–Whitney U test was used to compare two independent groups, while the Kruskal–Wallis test was used for comparisons involving more than two independent groups. Relationships between categorical variables were analyzed using Fisher’s Exact Chi-square test, and correlations between continuous variables were examined using Spearman’s Rho correlation coefficient. The predictive effects of independent variables (sarcopenia score, MNA score, gender, and mood) on the dependent variable, the frailty score, were evaluated through multiple linear regression analysis. A *p*-value of < 0.05 was considered statistically significant in all analyses.

### 2.4. Ethical Considerations of the Study

The study was approved by the Başkent University Institutional Review Board (Project No. KA19/264). Participants signed the Informed Consent Form, indicating that they voluntarily agreed to participate in the study. The study was conducted in accordance with the principles of the Declaration of Helsinki.

## 3. Results

Table 1 shows the mean scores and distribution of individuals’ frailty status according to the Edmonton Frailty Scale. The mean frailty scale score of the men was 6.1 ± 3.43, and that of the women was 8.8 ± 3.52. Frailty was more prevalent in women than men. The difference between gender was statistically significant (*p* < 0.05).

Frailty was found to be more prevalent in older adults, those who were single, had illnesses, used multiple medications, and had poor appetites (Table 2).

The frequencies of sarcopenia, mood, and nutritional status of the participants is shown in Table 3. According to SARC-F, the frequency of sarcopenia among the older population was 52.6% (65.4% in women, 46% in men). The frequency of exhaustion was 22.7% in non-frail participants and 94.2% in severely frail participants. A sad and depressed mood was reported by 18.2% of non-frail participants and 41.2% of severely frail participants. Among the severely frail participants, 35.3% were malnourished, 47.1% were at risk of malnutrition, and 17.6% had a normal nutritional status. There were statistically significant differences in sarcopenia status, exhaustion, and malnutrition based on the distribution of the frailty status among the participants (*p* < 0.05). Moderately and severely frail older adults were more likely to take nutritional supplements than the other groups.

In Table 4, the adequacy levels of dietary daily energy and nutrient intakes of the participants according to TUBER-2015 recommendations by frailty status are shown. The dietary protein and fat intake of all frail and non-frail groups was adequate, whereas dietary carbohydrate intake was inadequate in the majority of groups. The daily dietary intakes of fiber, vitamin D, vitamin B_1_, vitamin B_6_, and calcium were lower than recommended. There were statistically significant differences in vitamin C intake adequacy among groups (*p* < 0.05).

The correlations between frailty, sarcopenia, and nutritional status among the older adults is shown in Table 5. There was a positive correlation between frailty status and sarcopenia (r = 0.773; *p* < 0.05). Frailty and nutritional status were significantly negatively correlated (r = −0.496; *p* < 0.05). There was a significant positive correlation between the sarcopenia status and malnutrition status of the participants (r = 0.489; *p* < 0.005).

A multiple regression model analysis determining the impact of factors affecting frailty is shown in Table 6. A “Frailty = 2.869 + 0.749 × Sarcopenia − 0.364 × Malnutrition − 2.766 × Male − 0.951 × Energetic + 1.967 × Tired” model was estimated. According to this model, a one-unit increase in participants’ sarcopenia scores increased their frailty scores by 0.749 points. An increase in participants’ malnutrition scores was associated with a 36.4% decrease in their frailty scores. Men had a 76.6% lower frailty score than women. Participants who experienced exhaustion had frailty scores that were 1.967 times greater than those who felt normal. The effect of the parameters was statistically significant (*p* < 0.05). The effect of energetic mood on frailty was not found to be statistically significant (*p* > 0.05).

This model accounted for 76.7% of the total variance and was found to be statistically significant (*p* < 0.05).

## 4. Discussion

In this study, 22.4% of the older population were severely frail and 15.8% were moderately frail, and sarcopenia frequency was statistically significant according to the distribution of frailty status among individuals. In the North West Adelaide Health Study (mean age 74.1 (6.1) years, 55.5% female), 2.8% of 716 community-dwelling persons aged 65 years and older were both frail and sarcopenic, 15.5% were frail only, and 3.5% were sarcopenic only [24]. Sarcopenia has been reported in at least 1 in 20 individuals living in the community and in one third of the frail older adults living in nursing homes [7]. One study confirmed the hypothesis that frail people are at higher risk of sarcopenia and that sarcopenia is an important factor in the development of frailty [25]. In a study investigating the prevalence of geriatric syndrome in individuals over 60 years of age in Turkey, sarcopenia was found in 42% of frail individuals [25]. Petermann-Rocha et al. [26] also reported a strong association between frailty and sarcopenia in their study. In our study, a positive relationship was found between frailty and sarcopenia, similar to the results of other studies.

It has been noted that frailty and depression are two distinct entities, but they show a high degree of overlap in populations categorized as both frail and depressed due to similar responses to the same stressors [9]. In the present study, 31.8% of non-frail individuals, 50.0% of vulnerable individuals, 63.6% of mildly frail individuals, 83.3% of moderately frail individuals, and 70.6% of severely frail individuals reported having a sad and depressed mood. Depression has been shown to be positively associated with cognitive frailty and physical frailty [27] Bruce’s study [8] also found that depression results from frailty, stating that this is due to the fact that frailty and physical decline cause emotional distress such as hopelessness and worthlessness in individuals. Aine M. NiMhaolan et al. [28] reported that anxiety and depression scores were higher in the group of frail elderly adults in their study. Increasing frailty scores in older adults are thought to cause individuals to feel a loss of independence, productivity, and control over their lives, increasing their sense of fatigue.

Severe frailty and malnutrition are very common in older adults [29]. In a meta-analysis analyzing the association between malnutrition and physical frailty in community-dwelling older adults, Verlaan et al. [30] reported that two out of three malnourished community-dwelling adults were frail, 10% of the frail were malnourished, and that frailty and malnutrition were not interchangeable geriatric syndromes. While 35.3% of the severely frail older adults were malnourished, there was a significant negative relationship between frailty and malnutrition scale scores (*p* < 0.05). Boulos, Salameh, and Barberger-Gateau [31] showed that frail people were more likely to be malnourished and that malnutrition was associated with increased frailty. In their study, Kamo et al. [32] found that the mortality rate of severely frail and malnourished individuals who stayed in a nursing home for 1 year was associated with all-cause mortality from other causes in old age. A meta-analysis examining the association between the pre-frailty period and malnutrition showed that the prevalence of the pre-frailty period and the risk of malnutrition overlapped by 49.7% in the total population [33]. In a cohort study of rural older people, 64% of Lebanese patients diagnosed with frailty had poor nutritional status, while 36% had normal nutrition. In contrast, 90% of non-frail individuals were well-nourished and 1.8% were malnourished [34]. Controlling the process of frailty and maintaining a healthy diet are important for reducing the risk of disease, prolonging the quality of life, and maintaining functional independence in older adults [35].

As part of a multidimensional dynamic process, frailty syndrome is closely related to nutritional status. Inadequate energy intake, which is a common nutritional problem in the older adult population and is responsible for weight loss and reduced strength, has been associated with frailty [11]. Low energy intake can lead not only to loss of fat stores, but also to loss of muscle mass [36]. This study showed that 70.6% of severely frail individuals had an inadequate daily energy intake and that in all groups the daily dietary carbohydrate intakes were lower than recommended. In an Italian cohort study of 802 individuals, low energy intake (<21 kcal/kg) was associated with an increased risk of frailty [13]. Shikany et al. [37] found that non-frail men consumed a more energy-dense diet than frail men. A systematic review examining the impact of diet on frailty emphasized high protein intake in 5 of 19 studies and concluded that a lower risk of frailty was associated with good diet quality and high antioxidant capacity [38]. The Healthy ABC study showed that individuals with higher protein intakes had less loss of lean tissue mass after 3 years of follow-up [10]. Optimal protein intake in older adults is a complex issue. Adequate protein intake is reported to be necessary to prevent sarcopenia and frailty [39]. In this study the protein intake of all individuals was adequate.

In this study dietary fiber, vitamin D, vitamin B1, vitamin B6, and calcium intakes of older adults were lower than recommended, and did not differ statistically according to frailty status. In a study evaluating frailty and micronutrient intake, frail individuals were reported to have two or more micronutrient deficiencies compared to fit individuals [40]. Inadequate micronutrient intake has been associated with a higher risk of frailty in the older population. Although there has been a focus on vitamin D deficiency, other micronutrients have been suggested to play a role in the etiology of decreased physical activity and muscle strength with advancing age [41]. Low intakes or low plasma levels of carotenoids, selenium, magnesium, folate, vitamin C, vitamin E, n-3 fatty acids, and total polyphenols have been associated with frailty syndromes or frailty criteria [11]. Data from a cohort of more than 1600 individuals aged 65 years and older showed that lower intakes of vitamins B6, C, and E were associated with a higher risk of frailty [42]. While the impact of nutritional status on frailty has been examined in other studies, the impact of frailty on nutrition was examined in this study because it was believed that since the participants in this study were nursing home residents, the impact of frailty on nutrition would be mitigated by regular menu planning. Inadequate vitamin E intake has been associated with the loss of muscle strength and cognitive decline, which are features of frailty syndrome, and improvements in C-reactive protein and IL-6 levels have been observed in frail older adults with vitamin E supplementation [33]. In a study that examined the association between nutrient intake and frailty in 802 individuals over the age of 65, the risk of frailty was reported to be higher in individuals with inadequate dietary intake of vitamin E and vitamin C [13]. In this study, 94.1% and 76.5% of severely frail individuals had inadequate vitamin E and vitamin C intakes, respectively. There was a statistically significant difference in vitamin C intake adequacy according to the distribution of frailty status among participants (*p* < 0.05). This finding highlights the fact that poor dietary habits may have a negative impact on frailty, just as frailty may have a negative impact on nutritional status. In a study of 92 adults over the age of 75, the association between malnutrition, sarcopenia, and frailty in older nursing home residents was assessed. The assessment of the malnutrition status of the study participants using the MNA-SF showed that 33% were at risk of malnutrition, while the sarcopenia risk assessment using the SARC-F showed that one-third of the older adults were at risk, and 50% of the participants were pre-frail or frail according to the Frailty Index [43]. In this study there was a positive correlation between an individual’s frailty status and sarcopenia, and frailty and nutritional status were significantly negatively correlated (*p* < 0.05). A meta-analysis determining the frequency of frailty, sarcopenia, and malnutrition showed a 49.7% overlap between the pre-frailty period and malnutrition and a 41.6% overlap between sarcopenia and malnutrition risk [33]. In this study, frailty was positively associated with sarcopenia, female gender, and exhaustion, but negatively associated with malnutrition (*p* < 0.05). However, the effect of energetic mood on frailty was not found to be statistically significant (*p* > 0.05).

### Limitations and Strengths

There are some limitations in this study. The first limitation of this study is the small sample size and being a single-center study. Although Karaman, Turkey has a small population, all older adults living in nursing homes in the province and meeting the exclusion criteria were included in the study. The second limitation is that the relatively small sample size may have influenced the statistical power and the reliability of the conclusions drawn from the analyses. The third limitation is that the food consumption of the older adults was measured based on self-report, namely caregivers’ food records, rather than observation. In such cross-sectional studies, 24-h or 3-day 24-h food consumption records are usually taken to determine the nutritional status of individuals, while 7-day 24-h food consumption records were taken in this study. This is a strength of the study. Another strength of this study is that this is the first study in Turkey that investigates the relationship between frailty, sarcopenia, and nutritional status together in older adults living in nursing homes.

## 5. Conclusions

This is the first study to examine the relationship between frailty, malnutrition, sarcopenia, and nutritional status in older adults. By identifying the frailty status of older adults and assessing the impact of frailty on malnutrition, it is expected that older adults at risk can be better identified in advance, allowing the development of new strategies that will contribute to reducing unhealthy years.

## Figures and Tables

**Table 1:** Mean score and distribution of individuals according to Edmonton Frailty Scale.

Variables	Men (*n* = 50)		Women (*n* = 26)		Total (*n* = 76)		
	*n*	%	*n*	%	*n*	%	*p*-Value
Scale score ($\bar{X}\pm SD$)	6.1 ± 3.43		8.8 ± 3.52		7.0 ± 3.68		0.002 *
Score distribution							
Non-frail	18	36.0	4	15.4	22	28.9	
Apparently vulnerable	13	26.0	1	3.8	14	18.4	
Mildly frail	6	12.0	5	19.2	11	14.5	0.009 *
Moderately frail	6	12.0	6	23.1	12	15.8	
Severely frail	7	14.0	10	38.5	17	22.4	

* *p* < 0.05.

**Table 2:** Socio-demographical and general characteristics of individuals according to frailty status.

Frailty Status	Non-Frail		Apparently Vulnerable		Mildly Frail		Moderately Frail		Severely Frail		*p*-Value
	*n*	%	*n*	%	*n*	%	*n*	%	*n*	%	
Age (years) ($\bar{X}\pm SD$)	73.0 ± 8.18		76.9 ± 8.81		76.0 ± 6.27		75.8 ± 8.94		79.0 ± 6.30		0.207
Age groups, years											
65–74	14	63.6	6	42.9	5	45.5	6	50.0	4	23.6	0.188
75–84	5	22.8	5	35.7	6	54.5	3	25.0	10	58.8	
≥85	3	13.6	3	21.4	-	-	3	25.0	3	17.6	
Marital Status											
Married	3	13.6	3	21.4	3	8.3	1	8.3	2	11.8	0.705
Single	19	86.4	11	78.6	8	91.7	11	91.7	15	88.2	
Staying in nursing home, year											
<5	17	77.3	10	71.4	7	63.6	7	58.3	12	70.6	0.707
≥5	5	22.7	4	28.6	4	36.4	5	41.7	5	29.4	
Chronic disease											
Yes	16	72.7	9	64.3	10	90.9	12	100	17	100	0.010 *
No	6	27.3	5	35.7	1	9.1	-	-	-	-	
Drug use											
Yes	16	72.7	10	71.4	10	90.9	12	100	17	100	0.023 *
No	6	27.3	4	28.6	1	9.1	-	-	-	-	
Multiple drug use (3 and more)											
Yes	8	36.4	8	57.1	7	63.6	6	50.0	15	88.2	0.020 *
No	14	63.6	6	42.9	4	36.4	6	50.0	2	11.8	
Appetite											
Good	16	72.7	6	42.8	4	36.4	5	41.6	1	5.9	0.008 *
Normal	2	22.7	5	35.7	6	54.5	4	33.3	7	41.1	
Poor	1	4.5	3	21.4	1	9.1	3	25.0	9	53.0	

* *p* < 0.05.

**Table 3:** Sarcopenia, mood and nutritional status according to the frailty status of individuals.

	Frailty Status										*p*-Value
	Non-Frail		Apparently Vulnerable		Mildly Frail		Moderately Frail		Severely Frail		
	*n*	%	*n*	%	*n*	%	*n*	%	*n*	%	
Sarcopenia											
Yes	2	9.1	6	42.9	9	81.8	8	66.7	15	88.2	0.000 *
No	20	90.9	8	57.1	2	18.2	4	33.3	2	11.8	
Mood											
Energetic	6	27.3	-	-	-	-	-	-	-	-	0.000 *
Normal	11	50.0	8	57.1	4	36.3	2	16.6	1	5.8	
Exhausted	5	22.7	6	42.9	7	63.7	10	83.4	16	94.2	
Feeling sad and depressed											
Yes	7	31.8	7	50.0	7	63.6	10	83.3	12	70.6	0.029 *
No	15	68.2	7	50.0	4	36.4	2	16.7	5	29.4	
Nutrition Status (MNA)											
Malnourished	-	-	4	28.6	1	9.1	2	16.7	6	35.3	0.001 *
Risk of malnutrition	5	22.7	1	7.1	2	18.2	6	50.0	8	47.1	
Normal nutritional status	17	77.3	9	64.3	8	72.7	4	33.3	3	17.6	
Nutritional Supplement Use											
Yes	4	18.2	4	28.6	2	18.2	5	41.7	8	47.1	0.272
No	18	81.8	10	71.4	9	81.8	7	58.3	9	52.9	

* *p* < 0.05.

**Table 4:** The adequacy levels of dietary energy and nutrient intakes of older adults according to TUBER-2015 recommendations.

**Energy and Nutrients**	**Frailty Status**										***p*-Value**
	**Non-Frail**		**Apparently Vulnerable**		**Mildly Frail**		**Moderately Frail**		**Severely Frail**		
	***n***	**%**	***n***	**%**	***n***	**%**	***n***	**%**	***n***	**%**	
Energy, kcal											
Inadequate (F < 1502, M < 1867)	13	59.1	10	71.4	6	54.5	9	75	12	70.6	0.786
Adequate (F ≥ 1502, M ≥ 1867)	9	40.9	4	28.6	5	45.5	3	25	5	29.4	
Protein, %TE											
Inadequate (<12)	-	-	-	-	-	-	-	-	-	-	
Adequate (12–20)	22	100	14	100	11	100	12	100	17	100	
Fat, %TE											
Inadequate (<25)	-	-	-	-	-	-	-	-	-	-	
Adequate (25–30)	22	100	14	100	11	100	12	100	17	100	
Carbohydrate, %TE											
Inadequate (<45)	21	95.5	13	92.9	10	90.9	10	83.3	16	94.1	0.780
Adequate (45–60%)	1	4.5	1	7.1	1	9.1	2	16.7	1	5.9	
Fiber, g											
Inadequate (<25)	22	100	14	100	11	100	12	100	17	100	
Adequate (25–30%)	-	-	-	-	-	-	-	-	-	-	
Vitamin A, mcg											
Inadequate (F < 650, M < 750)	1	4.5	-	-	-	-	-	-	-	-	
Adequate (F ≥ 650, M ≥ 750)	21	95.5	14	100	11	100	12	100	17	100	
Vitamin D, mcg											
Inadequate (<15)	22	100	14	100	11	100	12	100	17	100	
Adequate (≥15)	-	-	-	-	-	-	-	-	-	-	
Vitamin E, mg											
Inadequate (F < 11, M < 13)	20	90.9	12	85.7	7	63.6	10	83.3	16	94.1	0.247
Adequate (F ≥ 11, M ≥ 13)	2	9.1	2	14.3	4	36.4	2	16.7	1	5.9	
Vitamin B_1_, mg											
Inadequate (<1.2)	22	100	14	100	11	100	12	100	17	100	
Adequate (≥1.2)	-	-	-	-	-	-	-	-	-	-	
Vitamin B_2_, mg											
Inadequate (F < 1.2, M < 1.3)	10	45.5	12	85.7	8	72.7	8	66.7	11	64.7	0.170
Adequate (F ≥ 1.2, M ≥ 1.3)	12	54.5	2	143	3	27.3	4	333	6	35.3	
**Nutrients**	**Frailty Status**										***p*-Value**
	**Non-Frail**		**Apparently Vulnerable**		**Mildly Frail**		**Moderately Frail**		**Severely Frail**		
	***n***	**%**	***n***	**%**	***n***	**%**	***n***	**%**	***n***	**%**	
Vitamin B_6_, mg											
Inadequate (F < 1.5, M < 1.7)	22	100	14	100	11	100	12	100	17	100	
Adequate (F ≥ 1.5, M ≥ 1.7)	-	-	-	-	-	-	-	-	-	-	
Total Folate, mcg											
Inadequate (<330)	20	90.9	14	100	11	100	12	100	16	94.1	0.803
Adequate (≥330)	2	9.1	-	-	-	-	-	-	1	5.9	
Vitamin B_12_, mcg											
Inadequate (<4)	3	13.6	3	21.4	1	9.1	2	16.7	2	11.8	0.933
Adequate (4–25)	19	86.4	11	78.6	10	90.9	10	83.3	15	88.2	
Vitamin C, mg											
Inadequate (F < 95, M < 110)	11	50	13	92.9	7	63.6	10	83.3	13	76.5	0.042 *
Adequate (F ≥ 95, M ≥ 110)	11	50	1	7.1	4	36.4	2	16.7	4	23.5	
Calcium, mg											
Inadequate (<950)	22	100	14	100	11	100	12	100	17	100	
Adequate (≥950)	-	-	-	-	-	-	-	-	-	-	
Magnesium, mg											
Inadequate (F < 300, M < 350)	22	100	14	100	11	100	12	100	17	100	
Adequate (F ≥ 300, M ≥ 350)	-	-	-	-	-	-	-	-	-	-	
Iron, mg											
Inadequate (<11)	21	95.5	13	92.9	11	100	12	100	16	94.1	0.825
Adequate (F: 11–16, M: 11)	1	4.5	1	7.1	-	-	-	-	1	5.9	
Zinc, mg											
Inadequate (F < 7.5, M < 9.4)	8	36.4	10	71.4	5	45.5	4	33.3	7	41.2	0.251
Adequate (F: 7.5–12.7, M: 9.4–16.3)	14	63.6	4	28.6	6	54.5	8	66.7	10	58.8	

TE: Total Energy F: Female M: Male. * *p* < 0.05.

**Table 5:** Correlations between frailty, sarcopenia and nutritional score.

		Frailty Score	Sarcopenia	MNA
Frailty Score	r	1	0.773	−0.496
	*p*		0.000 *	0.000 *
Sarcopenia	r	0.773	1	0.489
	*p*	0.000 *		0.000 *
MNA	r	−0.496	0.489	1
	*p*	0.000 *	0.000 *	

MNA: Mini Nutritional Assessment. * *p* < 0.05.

**Table 6:** Multiple linear regression analysis of variables affecting the frailty score.

Variables	ß (95% CI)	T	Coefficients *p*-Value	R^2^	Model *p*
Coefficient	2.869 (1.874–3.864)	5.748	0.0001 *	0.767	0.0001 *
Sarcopenia Score	0.749 (0.567–0.931)	8.206	0.0001 *		
Nutrition Status MNA	−0.364 (−0.511–0.216)	−4.917	0.0001 *		
Gender Male	−0.766 (−1.436–1.096)	−3.301	0.001 *		
Mood Energetic	−0.951 (−2.941–1.040)	−0.952	0.344		
Exhausted	1.967 (0.805–3.129)	3.374	0.001 *		

Frailty calculation; its predictors are summarized with confidence intervals and statistical significance values. MNA: Mini Nutritional Assessment * *p* < 0.05.

## Data Availability

The datasets generated and/or analyzed during the current study are not publicly available due to restrictions (e.g., they contain information that could compromise the privacy of research participants) but are available from the corresponding author (Serap İncedal Irgat, email: serapincedal@kmu.edu.tr) upon reasonable request.

## Abbreviations

SARC-F	Sarcopenia Rapid Screening Test
MNA	Mini Nutritional Assessment
